# Correlates of Inappropriate Prescribing of Antibiotics to Patients with Malaria in Uganda

**DOI:** 10.1371/journal.pone.0090179

**Published:** 2014-02-28

**Authors:** Arianna Rubin Means, Marcia R. Weaver, Sarah M. Burnett, Martin K. Mbonye, Sarah Naikoba, R. Scott McClelland

**Affiliations:** 1 Department of Global Health, University of Washington, Seattle, Washington, United States of America; 2 Department of Epidemiology, University of Washington, Seattle, Washington, United States of America; 3 Accordia Global Health Foundation, Washington DC, United States of America; 4 Infectious Diseases Institute, Makerere College of Health Sciences, Kampala, Uganda; 5 Department of Epidemiology and Social Medicine, University of Antwerp, Antwerp, Belgium; 6 Department of Medicine, University of Washington, Seattle, Washington, United States of America; The George Washington University Medical Center, United States of America

## Abstract

**Background:**

In many rural areas of Uganda, febrile patients presenting to health facilities are prescribed both antimalarials and antibiotics, contributing to the overuse of antibiotics. We identified the prevalence and correlates of inappropriate antibiotic management of patients with confirmed malaria.

**Methods:**

We utilized individual outpatient data from 36 health centers from January to September 2011. We identified patients who were prescribed antibiotics without an appropriate clinical indication, as well as patients who were not prescribed antibiotics when treatment was clinically indicated. Multivariate logistic regression models were used to identify clinical and operational factors associated with inappropriate case management.

**Findings:**

Of the 45,591 patients with parasitological diagnosis of malaria, 40,870 (90%) did not have a clinical indication for antibiotic treatment. Within this group, 17,152 (42%) were inappropriately prescribed antibiotics. The odds of inappropriate prescribing were higher if the patient was less than five years old (aOR 1.96, 95% CI 1.75–2.19) and if the health provider had the fewest years of training (aOR 1.86, 95% CI 1.05–3.29). The odds of inappropriate prescribing were lower if patients had emergency triage status (aOR 0.75, 95% CI 0.59–0.96) or were HIV positive (aOR 0.31, 95% CI 0.20–0.45). Of the 4,721 (10%) patients with clinical indications for antibiotic treatment, 521 (11%) were inappropriately not prescribed antibiotics. Clinical officers were less likely than medical officers to inappropriately withhold antibiotics (aOR 0.54, 95% CI 0.29–0.98).

**Conclusion:**

Over 40% of the antibiotic treatment in malaria positive patients is prescribed despite a lack of documented clinical indication. In addition, over 10% of patients with malaria and a clinical indication for antibiotics do not receive them. These findings should inform facility-level trainings and interventions to optimize patient care and slow trends of rising antibiotic resistance.

## Introduction

In Uganda, malaria is the leading cause of morbidity and mortality, accounting for 25–40% of outpatient visits to health facilities and nearly half of inpatient pediatric deaths [1]. In many rural areas, febrile patients presenting to health facilities are prescribed both antimalarials and antibiotics, contributing to the overuse of antibiotics [2]–[4]. While clinical algorithms such as the WHO's Integrated Management of Childhood Illness (IMCI) explicitly identify conditions appropriate for antibiotic treatment, the WHO estimates that in less developed countries only 40% of primary care patients in the public sector and 30% in the private sector are treated according to clinical guidelines [5], [6]. Overuse of antibiotics contributes to antimicrobial resistance, high healthcare costs, and poor patient confidence in healthcare quality [6]–[8].

Enhanced algorithmic trainings and guidelines such as the IMCI and the Integrated Management of Adult Illness (IMAI) have demonstrated utility in improving the quality of clinical care in low-resource countries [9]–[15]. However, increasing training coverage alone is not sufficient. In Uganda, while IMCI trained health workers deliver significantly better healthcare compared to their counterparts not trained in IMCI, absolute levels of service quality are still low [10]. A review of factors that influence IMCI adherence found that supervision, in-service training, and job aids gave mixed results in improving the use of medicines and health worker performance [16]. Thus, it is not well understood which clinical and operational factors are associated with health worker deviation from clinical guidelines and inappropriate drug treatment.

The objective of this study was to identify the prevalence and correlates of inappropriate antibiotic treatment in patients with a positive malaria smear or rapid diagnostic test (RDT), in a setting with endemic malaria and other bacterial diseases. Specifically, this analysis utilized data from the Uganda Infectious Diseases Capacity-Building Evaluation (IDCAP) mixed design cluster-randomized trial (CRT) to identify the clinical and operational factors associated with incorrect use of antibiotics, as well as failure to prescribe antibiotic treatment when clinically indicated. IDCAP analyses found that enhanced training interventions improved clinical performance in malaria case management. However the interventions did not influence overall antibiotic prescribing practices in patients with a positive malaria test [17]. This analysis will be helpful in understanding correlates of inappropriate treatment practices and in guiding the design of clinical trainings aimed at ensuring high quality care in low resource areas.

## Methods

### Study design and setting

In the IDCAP CRT two mid-level practitioners from each of 36 Ugandan level IV health centers (or comparable facilities) participated in a core Integrated Management of Infectious Disease (IMID) training course. The initial three week session was followed by two one-week booster courses from March to December 2010 [18]. All sites received the IMID intervention. Half of the sites were randomly selected to receive an additional two-day, on-site support (OSS) training, which included continuous quality improvement (CQI), each month from April to December 2010 (Arm A). The other half of the sites served as controls in 2010 and later received OSS monthly from November 2009 to September 2011 (Arm B). Standardized outpatient forms were used to collect data regarding facility-based indicators in all participating clinics during the follow-up period in 2011. The inclusion and exclusion criteria for facilities and health providers have been described in detail in an earlier IDCAP publication [18].

This study is a multi-level cross-sectional analysis of the IDCAP follow-up data with individual patients as the unit of analysis. Participants were male and female patients of any age who attended healthcare facilities from January through September 2011, and had a positive test for malaria by microscopic smear or RDT. These individuals accessed care at 36 health centers, representing all health administrative regions in Uganda. All observations were limited to the IDCAP study's follow-up period in 2011 because the primary research interest is correlates of inappropriate antibiotic prescribing rather than a comparison between the study arms. In addition, the quality of the triage data were better in 2011 than in 2009 and 2010 due to ongoing efforts to improve data quality. The effects of the IDCAP interventions could not be tested in an analysis of data from the follow-up time period alone.

### Identification of outcomes: Inappropriate antibiotic management

Malaria positive patients were categorized according to whether they had additional diagnoses necessitating antibiotic treatment. Diagnoses were recorded on standardized outpatient forms by health providers marking diagnosis checkboxes or writing diagnoses on the forms by hand. Diagnoses written by hand were manually screened and categorized. All diagnoses were classified according to whether antibiotic treatment would be considered appropriate or inappropriate according to the Ministry of Health's National Guidelines on Management of Common Conditions, the IMCI manual, and the IMAI manual [5], [19], [20]. Conditions that were considered to be appropriate versus not appropriate for antibiotic treatment were further reviewed by a group of Ugandan physicians to ensure that they were aligned with local standards of care. Diagnoses identified as appropriate and inappropriate indications for antibiotic treatment can be found in the supporting documents online (Table S1). Patients were classified according to whether or not they received an antibiotic without an appropriate clinical indication or, in contrast, did not receive an antibiotic when antibiotic treatment was clinically indicated. Even when no indication for antibiotic treatment was documented, administration of doxycycline was considered appropriate due to its utility in malaria treatment.

### Identification of clinical and operational exposures

Eight primary exposures were selected for analysis, including three clinical variables and five operational variables. Clinical exposure variables included HIV status, triage status, and age. Patients categorized as HIV positive only included individuals diagnosed as positive at the time of their study visit. HIV positive patients who received cotrimoxazole prophylaxis were not considered to have received inappropriate antibiotics, as this is the standard of care. Triage status was categorized by health providers on the standardized outpatient forms as emergency, priority, or standard. Age was categorized into three levels including less than 5 years, 5–14 years, and greater than 14 years of age.

Operational exposures are facility-level exposures that may influence a health provider's perception of risk, ability to treat patients according to clinical guidelines, and decision-making latitude. Exposures selected for this analysis included training level of the health provider, patient returning with the same chief complaint, less than 50% antimalarial availability during the week of a patient's visit, less than 50% antibiotic availability during the week of a patient's visit, and the entomological inoculation rate (EIR) associated with a health facility's coverage area. Health provider cadre, in order of decreasing years of training were medical officer, clinical officer, nurse, midwife, and other (less-skilled nursing assistants and other health professionals visiting the facility on a temporary basis, such as students). Patient visits were categorized as first visit for chief complaint versus two or more visits for the same primary complaint. Antimalarial availability was calculated by dividing the number of patients who actually received any antimalarial by the number of patients for whom an antimalarial was prescribed in a given week. Antibiotic availability was calculated in the same manner. The EIR is the number of infective bites per person per year, reported by the Ugandan MOH and categorized as very high, medium high, low, or very low [21].

### Statistical Analysis

We generated descriptive statistics by calculating number and percentage of the population for each categorical variable. Associations between exposure variables and the two outcomes of inappropriate antibiotic use and failure to prescribe antibiotics when clinically indicated were first evaluated using univariate logistic regression models. Variables associated with the outcomes in univariate analyses (P<0.1) were included in multivariate logistic regression models using a general estimating equation, clustering by health facility to account for intra-facility correlation. Separate multivariate models were fit to describe clinical and operational associations with inappropriate antibiotic treatment and with failure to prescribe antibiotics when clinically indicated. Model fit was assessed at both the univariate and multivariate levels using the link test. Residual analyses were conducted for each model by analyzing delta-beta values. The threshold for statistical significance was a two-tailed p-value≤0.05. Analyses were performed using Stata v.11 (Stata Corp, College Station, TX).

In addition to considering our primary exposures for inclusion in the multivariate models, three additional variables were identified *a priori* as potential confounding factors. These included facility type (private or public), patient gender, and visit month. The covariates were included in the multivariate model when the variable was associated with the outcome of interest in univariate analysis (P<0.1).

Multiple imputation was used to address missing values for five variables including age (0.7% missing), triage status (11% missing), provider training level (12% missing), return visits (3.5% missing), and patient gender (1.5% missing). The multivariable imputation via chained equations (MICE) method was used based on the assumption that data were missing at random. Fifteen variables were included in the imputation model. Logistic regression was used to impute missing values with five iterations. Derived estimates from each iteration were combined using Rubin's methods [22]. We also performed a sensitivity analysis including only complete cases. Estimates from the complete case analysis did not differ substantively from those produced with multiple imputation. Therefore, only the ORs, 95% confidence intervals, and p-values derived through imputation are presented. We performed a second sensitivity analysis to control for treatment arms and the potential influence of the IDCAP interventions.

The original IDCAP protocol was reviewed and approved by the Makerere University School of Medicine Research and Ethics Committee and the Uganda National Council on Science and Technology. The University of Washington Human Subjects Division determined that this secondary analysis of anonymous data did not meet the regulatory definition of research under 45 CFR 46.102(d). Anonymous data are available for public use and instructions for requesting them are on the Accordia Global Health Foundation website (http://www.accordiafoundation.org/IDCAP/data).

## Results

From January to September 2011, 45,591 patients tested positive for malaria by RDT or smear in the 36 health facilities participating in the IDCAP study. Of these patients, 25,800 (57%) were female (Table 1), 22,778 (50%) were under the age of five, and 1,109 (3%) were categorized as emergency triage status. Additionally, 33,783 (74%) of patients were visiting facilities in very high EIR areas, 19,972 (44%) of patients were seen by a clinical officer, and 737 (2%) of patients were seen by medical officers. Only 432 (1%) of patients had a positive HIV test at the time of their visit. There were 40,870 (90%) patients without a clinical indication for antibiotic treatment. Within this group 17,152 (42%) were inappropriately prescribed antibiotics. In contrast, of the 4,721 (10%) malaria patients with a clinical indication for antibiotic treatment, only 521 (11%) were inappropriately not prescribed an antibiotic.

**Table 1:** Characteristics of 45,591 Malaria Positive Patient Visits in Ugandan Clinics.

Characteristics	n	(%)
Indication for antibiotics	4,721	(10)
Prescribed antibiotics	21,352	(47)
IDCAP Intervention Arm^1^	20,637	(45)
Female	25,800	(57)
***Age***		
≤5 years	22,778	(50)
6–14 years	7,238	(16)
15+ years	15,250	(34)
***Clinical status***		
Standard triage status	35,401	(78)
Priority triage status	3,975	(9)
Emergency triage status	1,109	(3)
Underweight for age	210	(0.5)
HIV positive^2^	432	(1)
TB positive	19	(0.0)
***Process indicators***		
Antimalarial unavailability^3^	4,289	(9)
Antibiotic unavailability^3^	9,360	(21)
Very low EIR area^4^	462	(1)
Low EIR area^4^	1,603	(4)
Medium high EIR area^4^	9,743	(21)
Very high EIR area^4^	33,783	(74)
Multivitamin Treatment	1,510	(3)
ORS Treatment^5^	4,978	(11)
Repeat visit^6^	565	(1)
Medical officer	737	(2)
Clinical officer	19,972	(44)
Nurse	13,419	(29)
Midwife	752	(2)
Other (less skilled providers)	5,057	(11)

1 Infectious Diseases Capacity-Building Evaluation.

2 HIV test was positive at the patient's visit.

3 Drug was available 0–50% of all patient visits in a week.

4 Entomological inoculation rate of health facility visited.

5 Patients treated with oral rehydration solution.

6 Patient visited the facility two or more times for the same chief complaint.

### Antibiotic prescribing for patients without an indication for antibiotic treatment

In a univariate analysis of clinical factors associated with inappropriate prescribing of antibiotics to patients without an indication, those who were HIV-positive (OR 0.24, 95% CI 0.18–0.32) and those classified as emergency triage status (compared to standard; OR 0.63, 95% CI 0.55–0.71) were less likely to receive antibiotics in univariate analyses (Table 2). Compared to adults, children less than five years old (OR 1.94, 95% CI 1.86–2.03) and children 5–14 years old (OR 1.07, 95% CI 1.01–2.14) were more likely to receive antibiotics. In multivariate analysis controlling for confounding factors, HIV positive and emergency triage patients remained less likely to be inappropriately treated with antibiotics. Children under five remained more likely to be inappropriately treated relative to adults.

**Table 2:** Association between clinical exposure variables and inappropriate antibiotic treatment in patients without a clinical indication for antibiotics.

	Univariate Analysis			Multivariate Analysis		
Variable	OR	(95% CI)	p-value	OR^1^	(95% CI)	p-value
HIV-positive	0.24	(0.18–0.32)	0.01	0.31	(0.20–0.45)	<0.001
Standard triage status	*ref*	*ref*	*ref*	*ref*	*ref*	*ref*
Priority triage status	0.93	(0.80–1.07)	0.32	0.93	(0.71–1.20)	0.55
Emergency triage status	0.63	(0.55–0.71)	<0.001	0.75	(0.59–0.96)	0.02
Age 15+	*ref*	*ref*	*ref*	*ref*	*ref*	*ref*
Age 5–14	1.07	(1.01–1.14)	0.03	1.11	(0.98–1.26)	0.09
Age less than 5	1.94	(1.86–2.03)	<0.001	1.96	(1.75–2.19)	<0.001

1 Adjusted for patient gender, facility type (private or public) visited, and month of patient visit.

Considering operational factors associated with inappropriate prescribing of antibiotics, univariate analyses demonstrated that patients visiting two or more times for their chief complaint (OR 0.83, 95%CI 0.69–0.99) were less likely to be inappropriately treated with antibiotics (Table 3). Patients visiting facilities in low EIR areas (OR 0.81, 95% CI 0.73–0.90) were also less likely to be inappropriately prescribed an antibiotic. Nurses (OR 1.25, 95% CI 1.06–1.47), midwives (OR 1.27, 95% CI 1.02–1.59), and other providers with fewer years of training (OR 1.84, 95 CI%1.55–2.19) were more likely to inappropriately prescribe antibiotics relative to medical officers. Patients visiting facilities in medium high EIR areas (OR 1.25, 95% CI 1.19–1.31), during antimalarial shortages (OR 1.43, 95% CI 1.34–1.53), or during antibiotic shortages (OR 1.08, 95% CI 1.03–1.13) were also more likely to be inappropriately treated. In multivariate analysis, only care provided by lower skilled health providers and antimalarial shortages remained significantly associated with increased odds of inappropriate treatment.

**Table 3:** Association between operational exposure variables and inappropriate antibiotic treatment in patients without a clinical indication for antibiotics.

	Univariate Analysis			Multivariate Analysis		
Variable	OR	(95% CI)	p-value	OR^3^	(95% CI)	p-value
Return visit	0.83	(0.69–0.99)	0.04	0.82	(0.61–1.11)	0.21
Medical Officer	*ref*	*ref*	*ref*	*ref*	*ref*	*ref*
Clinical officer	1.10	(0.93–1.30)	0.27	1.15	(0.79–1.65)	0.46
Nurse	1.25	(1.06–1.47)	0.01	1.33	(0.85–2.09)	0.21
Midwife	1.27	(1.02–1.59)	0.04	1.29	(0.72–2.31)	0.39
Other health provider	1.84	(1.55–2.19)	<0.001	1.86	(1.05–3.29)	0.03
Very high EIR^1^	*ref*	*ref*	*ref*	*ref*	*ref*	*ref*
Medium high EIR^1^	1.25	(1.19–1.31)	<0.001	1.22	(0.68–2.20)	0.50
Low EIR^1^	0.81	(0.73–0.90)	<0.001	0.89	(0.55–1.42)	0.62
Very low EIR^1^	0.93	(0.76–1.14)	0.47	0.88	(0.43–1.81)	0.74
Antimalarial shortage^2^	1.43	(1.34–1.53)	<0.001	1.44	(1.02–2.01)	0.04
Antibiotic shortage^2^	1.08	(1.03–1.13)	0.002	0.96	(0.76–1.21)	0.75

1 Entomological inoculation rate.

2 Drug was only available 0–50% of all patient visits in a week.

3 Adjusted for patient gender, facility type (private or public) visited, and month of patient visit.

### Antibiotic prescribing for patients with an indication for antibiotic treatment

In patients with a clinical indication for antibiotic treatment, failure to prescribe antibiotics was considered to be a deviation from the clinical guidelines. In univariate analyses, those with priority (OR 0.30, 95% CI 0.19–0.48) or emergency (OR 0.33, 95% CI 0.23–0.46) triage status were less likely to have antibiotics withheld relative to standard triage patients (Table 4). In contrast, children under age 5 were more likely to have antibiotics withheld compared to adults (OR 1.38, 95% CI 1.14–1.68). However, in multivariate analysis, no clinical exposure variables remained significantly associated with the outcome of inappropriately withholding antibiotics.

**Table 4:** Association between clinical exposure variables and inappropriately withholding antibiotic treatment to patients with a clinical indication for antibiotics.

	Univariate Analysis			Multivariate Analysis		
Variable	OR	(95% CI)	p-value	OR^1^	(95% CI)	p-value
HIV-positive	0.29	(0.07–1.19)	0.09	0.32	(0.08–1.25)	0.10
Standard triage status	*ref*	*ref*	*ref*	*ref*	*ref*	*ref*
Priority triage status	0.30	(0.19–0.48)	<0.001	0.35	(0.11–1.11)	0.07
Emergency triage status	0.33	(0.23–0.46)	<0.001	0.43	(0.13–1.37)	0.15
Age 15+	*ref*	*ref*	*ref*	*ref*	*ref*	*ref*
Age 5–14	0.80	(0.55–1.17)	0.25	0.84	(0.57–1.23)	0.36
Age less than 5	1.38	(1.14–1.68)	0.001	1.35	(0.89–2.02)	0.16

1 Adjusted for patient gender, facility type (private or public) visited, and month of patient visit.

In univariate analyses of operational factors associated with failure to prescribe antibiotics when clinically indicated, patients visiting two or more times for their chief complaint were more likely to have treatment inappropriately withheld (OR 2.09, 95% CI 1.13–3.87) (Table 5). Patients visiting facilities in very low EIR (OR 2.07, 95% CI 1.07–4.00), low EIR (OR 1.40, 95% CI 0.74–2.66), and medium high EIR (OR 2.13, 95% CI 1.76–2.57) areas were also more likely to have treatment inappropriately withheld relative to patients visiting facilities in very high EIR areas. Midwives (OR 0.23, 95% CI 0.07–0.70) and other providers with lower training levels (OR 0.32, 95% CI 0.16–0.61) were less likely to inappropriately withhold antibiotics relative to medical officers. Clinical officers (OR 0.59, 95% CI 0.35–1.02) were also less likely than medical officers to inappropriately withhold antibiotics when a clinical indication was present, though this association was not statistically significant. In multivariate analysis, return patients and patients visiting facilities in medium high EIR areas remained more likely to have antibiotics withheld inappropriately. After adjustment for potential confounding factors, clinical officers were significantly less likely to inappropriately withhold antibiotics relative to medical officers (aOR 0.54, 95% CI 0.29–0.98).

**Table 5:** Association between operational exposure variables and inappropriately withholding antibiotic treatment to patients with a clinical indication for antibiotics.

	Univariate Analysis			Multivariate Analysis		
Variable	OR	(95% CI)	p-value	OR^3^	(95% CI)	p-value
Return visit	2.09	(1.13–3.87)	0.02	2.00	(1.09–3.69)	0.03
Medical Officer	*ref*	*ref*	*ref*	*ref*	*ref*	*ref*
Clinical officer	0.59	(0.35–1.02)	0.06	0.54	(0.29–0.98)	0.04
Nurse	0.60	(0.34–1.04)	0.07	0.59	(0.34–1.04)	0.07
Midwife	0.23	(0.07–0.70)	0.01	0.26	(0.06–1.09)	0.07
Other health provider	0.32	(0.16–0.61)	0.001	0.35	(0.16–0.76)	0.01
Very high EIR^1^	*ref*	*ref*	*ref*	*ref*	*ref*	*ref*
Medium high EIR^1^	2.13	(1.76–2.57)	<0.001	2.11	(1.52–2.94)	<0.001
Low EIR^1^	1.40	(0.74–2.66)	0.30	1.39	(0.94–2.04)	0.10
Very low EIR^1^	2.07	(1.07–4.00)	0.03	2.03	(0.65–6.35)	0.21
Antimalarial shortage^2^	1.24	(0.90–1.71)	0.19	**--**	**--**	**--**
Antibiotic shortage^2^	0.79	(0.62–1.00)	0.05	0.87	(0.55–1.36)	0.54

1 Entomological inoculation rate.

2 Drug was only available 0–50% of all patient visits in a week.

3 Adjusted for patient gender, facility type (private or public) visited, and month of patient visit.

To account for the potential effects of the IDCAP interventions a sensitivity analyses was conducted in which all multivariate analyses were additionally adjusted for IDCAP treatment arms. Because the ORs did not change significantly in these sensitivity analyses, the results presented are not adjusted for treatment arms.

## Discussion

In this nationwide sample of malaria patients presenting to health facilities in Uganda, over-prescription of antibiotics was extremely common. Specifically, while only 10% of patients were categorized as requiring antibiotic treatment according to clinical guidelines, 47% were prescribed an antibiotic. Despite the overall excess of prescribing antibiotics, there were also cases where antibiotics were withheld even when clinically indicated. In this sample, 11% of patients with indications for antibiotic treatment were not prescribed antibiotics. Considering these groups together, 39% of malaria patients did not receive appropriate antibiotic management according to clinical guidelines.

Our estimate of the proportion of malaria patients requiring antibiotic treatment is higher than estimates from an earlier study in Uganda [23]. In that study, it was estimated that approximately 5% of malaria patients required antibiotics based on clinical presentation, but 26% were prescribed antibiotics. Nonetheless, both studies show a substantial excess of antibiotic treatment. A review of 900 studies to identify patterns of antibiotic use in primary care found that 54% of patients in less developed countries were prescribed antibiotics [24]. This level of antibiotic use is similar to our finding that 47% of Ugandan malaria patients were prescribed antibiotics. However, most studies of antibiotic prescribing rates do not differentiate between appropriate and inappropriate use [25], [26].

Previous studies in Uganda and Tanzania found that malaria positive children under five are more likely to be prescribed antibiotics compared to older patients [23], [27]. This likely reflects provider concern regarding perceived patient vulnerability. Like the Tanzanian and Ugandan studies, our analyses showed that children under the age of five were more likely to be inappropriately prescribed antibiotics. In addition, our study adds important new information by demonstrating that much of the additional antibiotic treatment in malaria positive children under five is likely to be given despite the lack of a documented clinical indication.

Patients who were categorized as emergency triage status were less likely to be inappropriately administered antibiotics compared to standard triage patients. This finding may initially seem surprising, but could reflect greater provider confidence in malaria diagnoses when patients are both severely ill and have a positive malaria smear. It is also possible that more time is spent clarifying the diagnosis of patients with emergency triage status, increasing the likelihood of appropriate antibiotic use compared to patients who are not as urgently ill.

In our study, patients with an HIV-positive test result on the day of the visit who were prescribed cotrimoxazole were not considered inappropriately treated, since cotrimoxazole is recommended as prophylaxis for all HIV positive patients. We observed that patients with HIV were 70% less likely to be inappropriately prescribed antibiotics. It is possible that providers were more comfortable not prescribing additional antibiotics to patients already on cotrimoxazole.

In general, medical officers prescribed antibiotics less frequently than other health providers in our analysis. In the 90% of malaria patients with no clinical indication for antibiotics, medical officers were less likely than other health providers to inappropriately administer antibiotics. This may be due to the fact that clinical officers and other health providers with fewer years of training have high rates of antibiotic prescription, regardless of whether they are clinically indicated. A study of inappropriate antibiotic treatment of children with cough or diarrhea in Tanzania also found that medical officers were less likely to inappropriately treat children with antibiotics relative to health professionals with fewer years of training [28]. The authors reason that continuing medical education offered to higher-trained providers causes them to be less likely to engage in irrational antibiotic use. This may also be the case in our study, but requires further research. Of note, we also observed that medical officers were more likely to inappropriately withhold antibiotics when indicated relative to other health providers.

Malaria positive patients who visited health facilities during weeks when antimalarial availability was low (i.e. drugs were prescribed but not dispensed to more than half of patient visits that week) were more likely to be inappropriately prescribed an antibiotic. It is possible that when antimalarial stock was absent, providers felt compelled to prescribe another drug. Additionally, our definition of a drug shortage is influenced not only by the availability of drugs but by facility documentation of drug dispensing. Therefore, on busy days with high patient traffic, drug may have been prescribed and dispensed, but not recorded as such. Further research into prescribing practices in the setting of stock shortages could help to address this question and point to strategies for reducing inappropriate antibiotic prescription practices.

Given rising rates of antibiotic resistance globally and the considerable overlap in febrile illness presentation, it has become increasingly important to identify strategies to achieve rational antibiotic use in malaria endemic countries [29], [30]. To our knowledge, this is the first study to disaggregate appropriate from inappropriate antibiotic use among malaria patients. By identifying correlates of inappropriate antibiotic use, these findings should inform development of improved training in the correct use of clinical guidelines for the management of malaria patients. However, training protocols in Uganda might have a greater impact if targeted to clinical officers and nurses as opposed to medical officers. When utilizing these findings it is important to consider the relative contributions of different correlates of inappropriate antibiotic management. For example, there were relatively fewer patient visits to medical officers in this study population in comparison to clinical officers and nurses (737, 19,972, and 13,419 respectively).

There are potential limitations to this analysis. First, record keeping and data entry are often incomplete in busy health centers [30], but relatively few data were missing in this sample. In addition, checkboxes on standardized patient forms were not available for all bacterial illnesses, and some providers may have failed to write diagnoses onto the forms by hand. The variable for provider cadre could have been miscoded. Provider diagnosis and treatment discretion is also important, but is not captured in our analysis. All of these factors make it likely that some diagnoses and prescribing practices were incorrectly categorized. A second potential limitation relates to the fact that several studies have documented high rates of concurrent malaria and bacteremia in children, leading some authors to conclude that antibiotics should be prescribed liberally to children with severe malaria [31]–[34]. It is possible that such studies influenced the prescribing practices of our providers. In this context, it is important to note that in our study, any diagnosis of suspected bacteremia or sepsis on patient records was categorized as appropriate treatment. A third limitation of this study is the fact that data captured prescriptions rather than the ultimate patient outcomes of morbidity and mortality. As such, these data do not capture the range of potential effects of inappropriate treatment. Lastly, the anonymous manner in which the data were collected did not allow us to control for multiple visits to a health facility by the same individual. It is possible that patients visiting for a second time already had antibiotics prescribed at a prior visit.

Ensuring high quality care involves ending the misuse of unnecessary services as well as promoting the provision of essential services [35]. However, there has been a paucity of research regarding the factors that lead to inappropriate antibiotic use in less developed countries [25]. This analysis identifies the clinical and operational processes associated with deviation from clinical guidelines resulting in inappropriate antibiotic management of malaria positive patients in Uganda. There were distinct differences between factors associated with inappropriate antibiotic treatment in patients without a clinical indication versus factors associated with withholding antibiotics when they were clinically indicated. These findings provide important data for decision making regarding refinement of enhanced capacity building trainings and potential interventions to optimize patient care and slow rising trends of antibiotic resistance.

## Supporting Information

Table S1 (DOCX)
