# Supplementary material for: Correlates of Inappropriate Prescribing of Antibiotics to Patients with Malaria in Uganda
Source: PLoS One. 2014 Feb 28;9(2):e90179. doi: 10.1371/journal.pone.0090179 (PMC3938663; doi:10.1371/journal.pone.0090179)
Supplement: Table S1 — (DOCX) [file pone.0090179.s001.docx]

| **Online Supporting Information: Clinical Conditions** | |
| --- | --- |
| **Considered to be indications for antibiotic treatment** | **Considered not to be indications for antibiotic treatment** |
| Acute ear infection or mastoiditis | Abortion |
| Amoebiasis | Acute diarrhea |
| Infected animal or snake bites | Acute flaccid paralysis |
| Appendicitis | Adult cardiovascular conditions |
| Balanitis | Alcohol or drug abuse |
| Bartholinitis | Allergies |
| Blepharitis | Amenorrhea |
| Brucellosis | Chicken pox |
| Carbuncles | Cirrhosis |
| Cellulitis | Cough |
| Cervicitis^1^ | Cyst^4^ |
| Cholera ^2^ | Depression |
| Conjunctivitis | Dermatitis |
| Infected wounds, sores, and burns | Diabetes |
| Cystitis^1^ | Dysmenorrhea |
| Dacryocystitis | Dyspepsia |
| Dysentery | Dyspnea |
| Endocarditis | Epilepsy |
| Endometritis | Epistaxis |
| Furuncles | Folliculitis^4^ |
| Gonorrhea | Fungal infections |
| Impetigo | GI disorders- non infective |
| Leprosy | Glomerulonephritis |
| Lymphadenitis | Goiter |
| Lymphangitis | Guinea Worm |
| Mastitis | Hemorrhagic Fever |
| Meningitis (non-specific)^3^ | Hemorrhoids |
| Osteomyelitis | Hepatomegaly |
| Paronychia^3^ | Hernia |
| Pelvic inflammatory disease | Herpes |
| Peptic ulcer | HIV/AIDS^5^ |
| Periapical abcess | Hydrocele |
| Perichondritis | Hypertension |
| Pericoronitis | Impacted ear wax |
| Peritonitis^1^ | Intestinal worms |
| Persistent (chronic) diarrhea^3^ | Laryngitis^6^ |
| Pertussis | Malaria |
| Plague | Malnutrition |
| Pleurisy^3^ | Measles |
| Post-operative complications^3^ | Metrorrhagia |
| Pyelonephritis | Minor dental problems |
| Pyoderma (not pyoderma gangrenosum) | Minor eye problems |
| Pyomyositis | Mumps |
| Rheumatic heart disease | Musculoskeletal conditions |
| Salmonellosis^3^ | Lymphoma |
| Severe Pneumonia | Neuropathy |
| Shigellosis | Obstructed labor |
| Sinusitis^3^ | Odynophagia |
| Staphylococcus and streptococcus infections | Oesophagitis |
| STI (non-specific)^3^ | Onchocerciasis |
| Syphilis | Orchitis **^6^** |
| Tetanus | Pain |
| Trachoma | Palpitations |
| Trichomoniasis | Pancreatitis |
| Typhoid | Papular pruritic eruption |
| Urethral discharge^1^ | Parotitis |
| Urethritis^1^ | Pemphigus |
| Vaginitis^3^ | Perinatal conditions in newborns |
|  | Pertussis |
|  | Phimosis |
|  | Pharyngitis |
|  | Polyps |
|  | Prurigo |
|  | Psoriasis |
|  | Rabies |
|  | Renal diseases |
|  | Respiratory tract infection**^7^** |
|  | Rheumatism |
|  | Scabies |
|  | Schistosomiasis |
|  | Sleeping sickness |
|  | Spondylitis |
|  | Stomatitis |
|  | Trauma (non-specified) |
|  | Tuberculosis ^8^ |
|  | Tumor or cancer |
|  | Ulcers (non-specified) |
|  | Uritcaria |
|  | Viral infection (non-specified) |
|  | Vitiligo |
|  | Yellow Fever |

^1^ Although the condition can be caused by a number of pathogens, bacterial causes are most common
^2^ Because severity has not been recorded in patient records, all cases are considered indications for antibiotic treatment

^3^ Because diagnoses are not specified by pathogen all cases are considered indications for antibiotics

^4^ Unless specified as infected or necrotic, in which case antibiotic treatment is indicated

^5^ Cotrimoxazole should be provided as part of comprehensive HIV care, but HIV/AIDS in of itself is not an indication for other antibiotic treatment during standard visits

^6^ Although disease can be bacterially associated, disease is most commonly virally induced

^7^ Other than those specified in the left hand column

^8^ TB medications are not considered standard antibiotics
